# Quantitative Aortic Distensibility Measurement Using CT in Patients with Abdominal Aortic Aneurysm: Reproducibility and Clinical Relevance

**DOI:** 10.1155/2017/5436927

**Published:** 2017-04-18

**Authors:** Yunfei Zha, Gongling Peng, Liang Li, Chunying Yang, Xuesong Lu, Zhoufeng Peng

**Affiliations:** ^1^Department of Radiology, Renmin Hospital of Wuhan University, Wuhan, China; ^2^Department of Thyroid and Breast Surgery, The Central Hospital of Wuhan, Tongji Medical College, Huazhong University of Science and Technology, Wuhan, China; ^3^Department of Radiology, Huaihe Hospital of Henan University, Kaifeng, China; ^4^Department of Biological Engineering, School of Biomedical Engineering, South-Central University for Nationalities, Wuhan, China; ^5^Hubei Key Laboratory of Medical Information Analysis & Tumor Diagnosis and Treatment, Wuhan, China

## Abstract

*Purpose.* To investigate the reproducibility of aortic distensibility (*D*) measurement using CT and assess its clinical relevance in patients with infrarenal abdominal aortic aneurysm (AAA).* Methods.* 54 patients with infrarenal abdominal aortic aneurysm were studied to determine their distensibility by using 64-MDCT. Aortic cross-sectional area changes were determined at two positions of the aorta, immediately below the lowest renal artery (level 1.) and at the level of its maximal diameter (level 2.) by semiautomatic segmentation. Measurement reproducibility was assessed using intraclass correlation coefficient (ICC) and Bland-Altman analyses. Stepwise multiple regression analysis was performed to assess linear associations between aortic *D* and anthropometric and biochemical parameters.* Results.* A mean distensibility of *D*_level  1._ = (1.05 ± 0.22) × 10^−5^  Pa^−1^ and *D*_level  2._ = (0.49 ± 0.18) × 10^−5^  Pa^−1^ was found. ICC proved excellent consistency between readers over two locations: 0.92 for intraobserver and 0.89 for interobserver difference in level 1. and 0.85 and 0.79 in level 2. Multivariate analysis of all these variables showed sac distensibility to be independently related (*R*^2^ = 0.68) to BMI, diastolic blood pressure, and AAA diameter.* Conclusions.* Aortic distensibility measurement in patients with AAA demonstrated high inter- and intraobserver agreement and may be valuable when choosing the optimal dimensions graft for AAA before endovascular aneurysm repair.

## 1. Introduction 

Abdominal aortic aneurysm (AAA), a progressive expansion and weakening of the abdominal aortic wall, is a common and potentially lethal vascular disease [1]. In the United States, there are approximately 15,000 deaths every year related to the rupture of AAA, a mortality rate that rivals that of ovarian cancer or leukemia [2]. According to the biomechanical approach in predicting the risk of aneurysm rupture and to the fundamental principles in cardiovascular mechanics, rupture occurs when the AAA wall stress exceeds the failure strength of the wall. Therefore, it becomes obvious that the knowledge of the distribution of distensibility on a particular AAA wall would be a good indication of its susceptibility to rupture [3, 4].

Distensibility and pulse wave velocity (PWV) are frequently used to evaluate arterial elasticity and to predict the risk of cardiovascular disease [5]. Aortic distensibility, relative change in volume per change in pressure or relative change in cross-sectional area per change in pressure, reflects the structural arrangement of the artery (particularly its elastic components). Animal studies suggest that reduced arterial elasticity is an early sign of atherosclerotic change [6]. PWV is the rate of propagation of the flow or pressure wave in an artery and inversely related to vascular distensibility. Hence, a stiffer vessel will conduct the pulse wave faster than a more distensible and compliant vessel.

Ultrasonographic screening for AAA is a technically simple diagnostic test that is associated with a major reduction of AAA-related mortality [7, 8]. However, preoperative assessment in obese patients is very difficult, and its accuracy can be significantly altered by visceral fat and bowel gasses, especially by the operators; therefore detecting aneurysm change with ultrasound is often unpredictable and highly variable. Recent studies have indicated that CT could be well used to assess area and distensible changes in animal or human aorta [9–13]. A strength of CT is the unique ability to provide both local and regional noninvasive measurement of aortic morphology.

Therefore, the aims of our study were to investigate the reproducibility of aortic distensibility measurement using CT and assess its clinical relevance in patients with infrarenal abdominal aortic aneurysm.

## 2. Material and Methods

### 2.1. Patients

Between March 2011 and February 2016, a total of 110 subjects presenting with AAA were prospectively recruited on referral for cardiovascular assessment in relation to consideration for endovascular aortic repair (EVAR). The decision to subsequently operate or not upon a patient in this study was performed according to the American College of Cardiology/American Heart Association Task Force on Practice Guidelines. The study received approval from the ethical committee of our hospital. The subjects provided fully informed consent to participate in this study by signing a written consent form. The inclusion criteria were the following: (i) presentation for investigation of AAA after ultrasound scan or strong clinical suspicion; (ii) initial aortic CTA measures a maximal axial infrarenal aortic diameter of >3 cm; and (iii) no aortic surgical history.

The exclusion criteria were as follows: (i) refusal to participate; (ii) contraindication to CTA, such as abnormal serum creatinine and contrast allergy; and (iii) aneurysm necks angulate (>60°) or necks < 1.5 cm long, for they were deemed noncandidates for EVAR [14]. All patients were not under beta-blocker therapy or received oral beta-blocker before examination.

### 2.2. CT Acquisition Protocol

Data were acquired on a 64-detector VCT system (General Electric Medical Systems, Milwaukee, USA). Acquisitions were obtained after an antecubital intravenous injection of 65–90 ml iopromide 370 (370 mg I/100 ml, Ultravist 370; Bayer Schering Pharma, Berlin, Germany) with 30–40 ml saline solution as bolus chaser at 4-5 ml·s^−1^. The targeted imaging region was the infrarenal abdominal aorta, which is where nearly 90% of aortic aneurysms occur [15]. Helical scans with pitch of 0.2 : 1 were acquired in a craniocaudal direction, with a retrospective ECG-gating on the R peaks of an ECG signal. The automated bolus tracking technique implemented on the scanner was applied, and a ROI was placed in the abdominal aorta using a threshold of 200 Hounsfield units (HU). Detector width was 40 mm, detector collimation was 0.625 mm, and reconstructed slice thickness was 0.625 mm. Gantry rotation time was 0.35 seconds. The maximum tube current ranged from 250 to 750 mA, depending on patient size, with a fixed tube voltage of 120 kVp.

#### 2.2.1. Data Processing and Software

**Figure 1:**
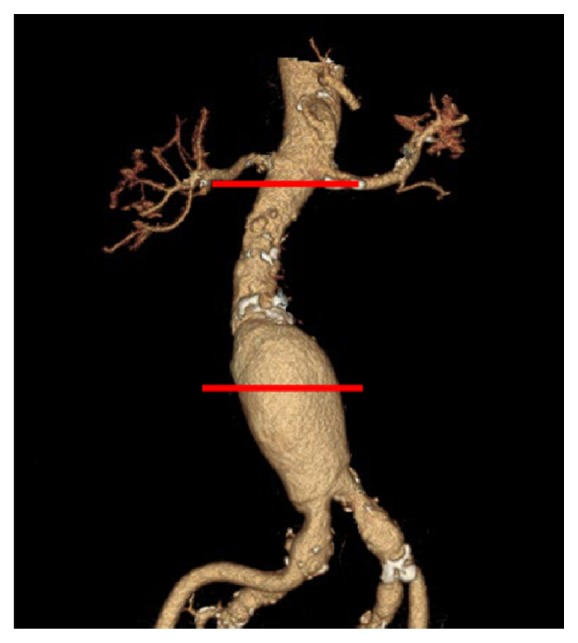
Volume rendering image. A 63-year-old man with infrarenal abdominal aortic aneurysm. The two positions immediately below the lowest renal artery (level 1.) and at the level of maximal aneurysm diameter (level 2.) for functional image reconstruction indicated as horizontal lines.

*Preprocessing. *CT datasets were reviewed on an advanced processing workstation (Advantage Windows Workstation, version 4.5 with CardIQ software, GE Healthcare). From the so derived dataset, 20 time frames per heart cycle were calculated at each position; that is, images were reconstructed at 0%, 5%, 10%,…, 95% of the R-R interval by 5%. Reconstruction slice thickness was 0.625 mm for the time resolved as well as for the morphological images. Aortic cross-sectional area changes were determined at two positions of the aorta, immediately below the lowest renal artery (level 1.) and at the level of its maximal diameter (level 2.) (Figure 1). The positions were selected by a senior radiologist experienced in vascular CT.

Two readers independently segmented the area of the aorta. They were a fourth-year resident (observer: A) in radiology with 1-year experience in cardiothoracic CT imaging and a sixth-year medical student (observer: B). To determine the interobserver reliability, in the first measurement, observation A1 was compared to observation B. For the intraobserver reliability, observation A2 by observer A in the second measurement after 2 weeks was compared with A1. All readers were blinded to all clinical data, including the observations from other readers.

Brachial arterial blood pressure was registered directly after image acquisition using an arm cuff. To minimize the error in pressure estimation, we measured blood pressure five times, using a validated automated artery sphygmomanometer (OmRon HEM-7201, OmRon Company, DaLian, China). These measurements were averaged to determine the mean pulse pressure of each subject.

**Figure 2:**
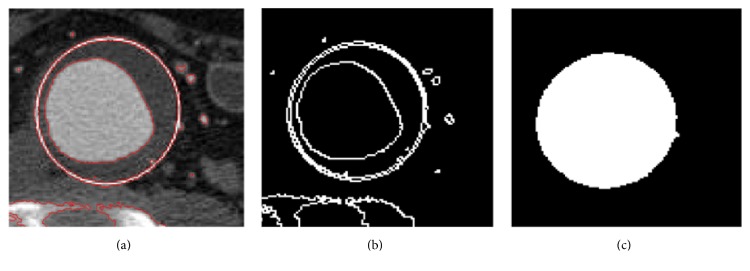
Processing steps required to calculate vessel area, shown for an example measurement at aneurysm sac level. The reconstructed CT images are segmented using the active contour algorithm (a), setting a seed point, and defining two ROIs in between which the vessel wall is found (b and c).

*Matlab Analysis*. The vessel areas were determined from the 20 images reconstructed within a given cardiac cycle in one scan. Since manual outlining of the aortic wall area was a very time-consuming and operator-dependent process, a semiautomatic segmentation algorithm was used to detect the arterial wall in all CT images. The algorithm has been described in the literature and tested previously [11, 16]. Briefly, the raw projection data were transferred to a standard PC via a local network. On this PC, all image data were analyzed by using programs developed on Matlab 7.5 (Version R2007b, the MathWorks, Natick, MA, USA) and Visual C++ (Microsoft, Redmond, WA, USA). In order to better fit the boundary, the final contour was refined by an iterative process (Figure 2).

*Distensibility Quantification. *In the literature, vascular distensibility (*D*) is defined as the relative change in vessel cross-sectional area that occurs during the cardiac cycle, divided by the corresponding change in blood pressure [11–13, 17]. Therefore, distensibility was calculated as the capacity of an artery to augment its area in relation to increasing intra-arterial pressure:

(1) $$\begin{array}{c} D\;\left[\;Pa^{-1}\;\right]=\frac{\Delta A}{A_{min}\cdot \Delta p}, \end{array}$$

where *A*_min_ is the minimum vessel area over the cardiac cycle and Δ*A* is the difference between maximum and minimum area. Pulse pressure (Δ*p*) value was defined as systolic minus diastolic blood pressure and can be estimated using sphygmomanometry. In order to compare results with literature values, distensibility was also converted into pulse wave velocity (PWV) [m/s]:

(2) $$\begin{array}{c} PWV\;\left[\;m/s\;\right]=\frac{1}{\sqrt{D\cdot \rho }}=\frac{1}{\sqrt{D\;\left[Pa^{-1}\right]\cdot 1055}}. \end{array}$$

Here, *ρ* = 1055 × 10^3^ [kg/m^3^] denotes the mass density of blood that is assumed to be constant.

For each time series the segmentation process was repeated three times, and the median cross-sectional area was used for the final analysis.

### 2.3. Statistical Analysis

All data were presented as mean value ± standard deviation, unless stated otherwise. Stepwise multiple regression analysis was performed to assess linear associations between aortic wall parameters as the dependent variables and determinants of clinical and cardiovascular parameters, including age, sex, body mass index (BMI), systolic blood pressure (SBP), diastolic blood pressure (DBP), and heart rate. Wilcoxon signed rank test was performed to assess the statistical differences at level 1. and level 2. Intra- and interobserver agreement was assessed using intraclass correlation coefficient (ICC) analysis and Bland-Altman method. Bland-Altman tests were performed with GraphPadPrism (GraphpadInstat, version 5.01 GraphPad Software, San Diego, California, USA). All other statistical analyses were performed using SPSS statistical package (SPSS for Windows, version 13.0; SPSS, Chicago, IL, USA). A *P* value of <0.05 (two-tailed) was considered significant.

## 3. Results

Of the 110 subjects enrolled, 54 (52%) subjects with AAA met criteria for inclusion into the study. Mean abdominal aortic size was 3.9 cm (range: 3.2–5.8 cm), measured with conventional US. The clinical characteristics of the population collected for this study are listed in Table 1. The mean age was 67.23 years (range: 50–76 years).

The ECG-gated CT images showed a high contrast between the contrast medium inside the vessel and the surrounding fatty tissue. With the active contour algorithm, the cross-sections of the aorta were segmented successfully in all 54 patients, and distensibility values and pulse wave velocities were calculated. No adverse events were recorded.

A mean distensibility of *D*_level  1._ = (1.05 ± 0.22) × 10^−5^ Pa^−1^ was found immediately below the lowest renal artery and *D*_level  2._ = (0.49 ± 0.18) × 10^−5^ Pa^−1^ at the level of maximal aneurysm diameter, which corresponds to pulse wave velocities of PWV_level  1._ = (9.68 ± 1.09) m·s^−1^ and PWV_level  2._ = (14.96 ± 4.01) m·s^−1^, respectively. Distensibility value in level 2. was significantly lower than one in level 1. in patients with AAA (*P* < 0.01). Summary of distensibility measurements is shown in Table 2.

**Figure 3:**
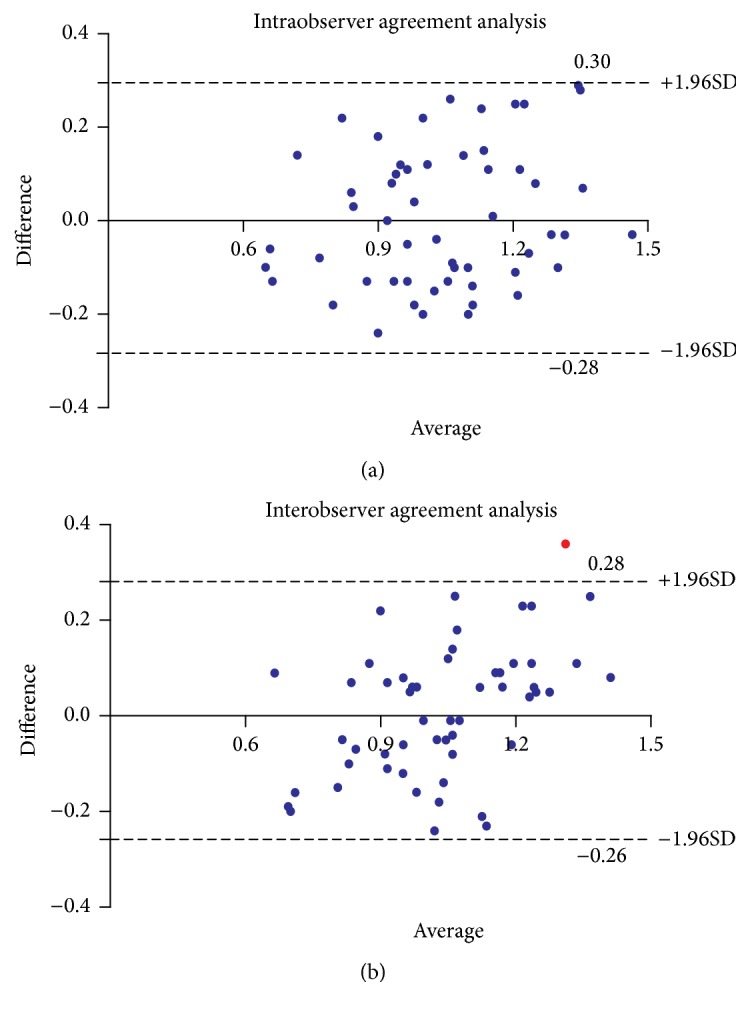
Bland-Altman plots show close degree of intra- and interobserver agreement of distensibility measurements immediately below the lowest renal artery. Upper and lower dashed lines = 95% CI for mean difference (±1.96 standard deviations). (a) The mean difference in measurement pairs was 0.017 × 10^−5^ Pa^−1^ (−0.28 to 0.30). (b) The mean difference in measurement pairs was 0.013 × 10^−5^ Pa^−1^ (−0.26 to 0.28).

**Figure 4:**
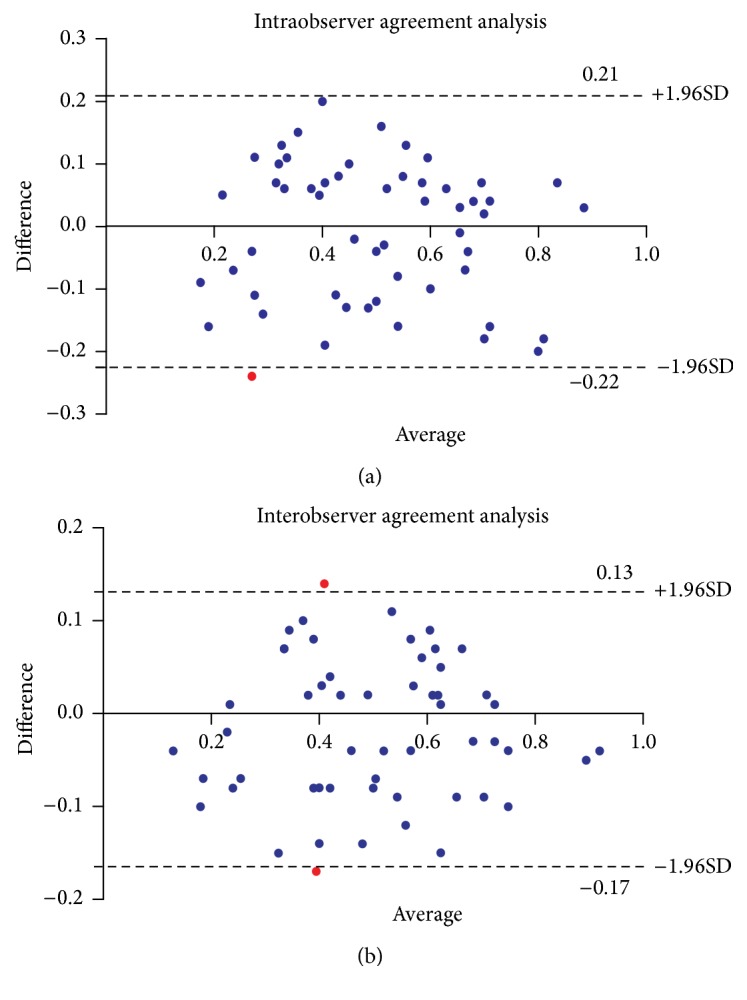
Bland-Altman plots show close degree of intra- and interobserver agreement of distensibility measurements at the level of maximal aneurysm diameter. Upper and lower dashed lines = 95% CI for mean difference (±1.96 standard deviations). (a) The mean difference in measurement pairs was 0.010 × 10^−5^ Pa^−1^ (−0.22 to 0.21). (b) The mean difference in measurement pairs was 0.018 × 10^−5^ Pa^−1^ (−0.17 to 0.13).

ICC results showed excellent consistency between readers over two locations: 0.92 (*P* < 0.01) for intraobserver and 0.89 (*P* < 0.01) for interobserver difference in level 1. and 0.85 (*P* < 0.01) and 0.79 (*P* < 0.01) in level 2. The mean intraobserver difference of distensibility in level 1. and level 2. was 0.017 × 10^−5^ Pa^−1^ (upper limit of agreement, 0.30 × 10^−5^ Pa^−1^; lower limit of agreement, −0.28 × 10^−5 ^Pa^−1^) and 0.010 × 10^−5^ Pa^−1^ (upper limit of agreement, 0.21 × 10^−5^ Pa^−1^; lower limit of agreement, −0.22 × 10^−5^ Pa^−1^), respectively. The mean interobserver difference in level 1. and level 2. was 0.013 × 10^−5^ Pa^−1^ (upper limit of agreement, 0.28 × 10^−5^ Pa^−1^; lower limit of agreement, −0.26 × 10^−5^ Pa^−1^) and 0.018 × 10^−5^ Pa^−1^ (upper limit of agreement, 0.13 × 10^−5^ Pa^−1^; lower limit of agreement, −0.17 × 10^−5^ Pa^−1^), respectively. Intraobserver agreement was better than interobserver agreement for distensibility measurements investigated in level 1. and level 2. This was also reflected by tighter 95% limits of agreement when compared with those obtained for the interobserver measurements. Data on intraobserver and interobserver variability were summarized in Table 3. The corresponding Bland-Altman plots were displayed in Figures 3 and 4.

The correlation coefficients between the anthropometric, biochemical values and aortic distensible parameters in AAA patients are shown in Table 4. Aortic sac distensibility was positively related to sex and height whereas it was inversely related to age, BMI, SBP, and DBP, AAA diameter, glucose, cholesterol, triglycerides, HDL, and LDL. Arterial distensibility was significantly lower in women, even if indexed to BMI. Multivariate analysis of all these variables showed sac distensibility to be independently related (*R*^2^ = 0.68) to BMI, DBP, and AAA diameter.

## 4. Discussion

This study demonstrated the potential value of an integrated CT protocol for comprehensive evaluation of aortic function in patients with AAA. The main findings in our study were that aortic sac stiffening as characterized by aortic distensibility and PWV was significantly increased as compared to the nonaneurysmatic parts of the self-control aorta. In addition, aortic sac stiffening correlated positively with BMI, DBP, and AAA diameter.

Interventional radiologists and vascular surgeons may be very interested in this study when choosing the optimal dimensions graft for AAA before EVAR. In general, EVAR require extensive preoperative assessment for aortic characteristics in order to select suitable graft. Especially for infrarenal AAA, the area just below the renal arteries, the distensibility is very important for adequate sealing of the proximal stent-graft attachment system. Appropriate sealing determines the success and durability of EVAR. It has recently been shown that the measurement of arterial distensibility is a significant predictor of rupture risk independently of aneurysm diameter [4, 18]. Therefore, precise characterization of aortic baseline and follow-up dimensions will be crucial in studies aimed at assessing the wall morphological and functional differences before EVAR.

Decreased aortic distensibility and increased PWV may be one of the contributors to arterial hypertension and subsequent cardiovascular complications. Blood pressure could function as both cause and effect: increased PWV and decreased distensibility increase systolic blood pressure, whereas increased blood pressure contributes to decreased arterial elastic properties. In other words, reduced arterial strain and elevated blood pressure may be mutually causally related. However, the potential of aortic distensibility as a reliable predictor of the risk of progression to AAA in large sample subjects still needs further investigation.

BMI is another important predictor of cardiovascular events in the general population, independent of blood pressure. Recently, a large population study of 12, 203 men confirmed that the central obesity independently associates with AAA formation [19]. A predominant localization of visceral abdominal fat may be relevant as perivascular adipose tissue which has been observed to affect inflammation and formation of experimental AAA in animals [20–22]. In addition to this, the AAA diameter decreased after weight loss in mice, which seemed to limit progression of the AAA sac growth [23]. In particular, obesity is becoming a more important determinant of vascular disease than blood lipids, at least in the present study population. These observations emphasize the importance of population-wide strategies directed to the reduction of levels of adiposity by a combination of changes in diet and physical activity.

In our present study, mean BMI was (29.7 ± 2.1) kg/m^2^ and distensibility value was (1.05 ± 0.22) × 10^−5^ Pa^−1^ below the lowest renal artery and (0.49 ± 0.18) × 10^−5^ Pa^−1^ at the level of maximal aneurysm diameter, which corresponds to PWV of (9.68  ±  1.09) m·s^−1^ and (14.96  ±  4.01) m·s^−1^, respectively. Using the same methods, Ganten et al. [13] found a mean distensibility of (1.3 ± 0.8) × 10^-5 ^Pa^−1^ and (0.6 ± 0.5) × 10^−5^ Pa^−1^ for two same locations in a AAA group of (68 ± 9)-year-old nonobese subjects. Using different ultrasound methods in AAA sac level, Kadoglou et al. [24] found a mean PWV of (12.99 ± 3.75) m·s^−1^ in 108 AAA patients of BMI = (28.98 ± 4.23) kg/m^2^, and Li et al. [25] found a mean PWV of (10.54 ± 6.52) m·s^−1^ in 8 AAA subjects without obesity. By comparing the absolute values derived from our study and that from previous publication, we found that the distensibility *D* (or PWV) was significantly lower (or higher) in our study. The reasons for this are not known but an excess of abdominal visceral fat has been found in our study population, which may be linked to the altered vascular function.

The significant morphological and functional differences were found in AAA at the point just below the lower branches of renal artery and the widest diameter of aneurysm, in the transversal diameter plane. The distensibility was significantly lower in the aneurysms than in the nonaneurysmatic parts of the aorta, in concordance with the study of Ganten et al. This result demonstrated that the proximal aorta was more distensible, whereas the wall of the aneurysm sac was stiffer. A publication by Mladenović et al. [26] described that, in a stiffened vessel wall, the impact of distending forces was more likely to cause damage than in a compliant wall, ultimately leading an aneurysm in this area. Elastic properties of the aortic wall are not constant with increasing stretch. Under normal physiological pressure load, the deformation with pressure is accommodated by the elastic lamina. Increasing distending pressure can exceed the elastic lamina's range of deformation [27]. Further pressure change beyond this was accommodated by inelastic collagen with a much lower *D*.

To our knowledge this is the first study investigating the inter- and intraobserver reproducibility for distensibility measurements in patients with AAA. Bland-Altman plots demonstrated excellent agreement between pairs of measurements made by the same observer (A1 and A2) and by the two different observers (A1 and B). Meanwhile, an interesting finding was that intraobserver agreement was better than interobserver agreement for distensibility measurements investigated in level 1. and level 2. A number of reasons may account for this phenomenon. The software used for this study is sometimes unable to automatically define the centerline at abdominal aorta, and manual centerline definition and adjustment may be necessary. In addition, the presence of atheromatous disease in the aortic wall, intraluminal thrombus (ILT), or the presence of an calcified plaque may result in erroneous centerline identification, requiring manual editing. Such manual adjustments may lead to variations in area measurements between different observers. Furthermore, measurement variability using semiautomatic centerline analysis may arise from differences in defining the outer contours of the aortic wall; occasionally these contours are difficult to ascertain and definition is somewhat arbitrary, particularly if contours are obscured by motion artifact or streak artifact from contrast. Thus, any errors due to technical limitations are greater for interobserver agreement.

The findings of the present study must be interpreted in the context of the following limitations. Our study did not take into consideration the ILT influence on AAA wall stress, which is a major limitation in this work. Given that for the wall distensibility estimation the pressure difference that acts on the wall is necessary, the assumption that the pulse pressure acts on the wall can be inappropriate. This is because the presence of thrombus can alter the wall stress loading. Specifically, Meyer et al. [28] have shown that thrombus can have a stress-reducing role even if it does not directly reduce pressurization of the wall, when thrombus is assumed to be fully attached to the wall. It has recently been shown that the wall distensibility, as measured by sac volume change and pulse pressure, should be corrected using a correction factor that includes thrombus percentage in the sac [29]. However, its exact role in AAA wall stress has been controversial. Other studies have proven that examination of ruptured AAAs has shown the presence of ILT at the location of failure [30]. It has been proposed that the presence of ILT induces hypoxia thus weakening the aneurismal wall and contributing to AAA rupture [31]. Secondly, due to the use of small helical pitch, our cardiac CT protocol was associated with a considerable CT dose index value. We have therefore chosen relatively short scan range to limit the total radiation dose to patients. This was acceptable since we were only interested in the maximum and minimum vessel areas and not in an exact temporal correlation within the heart cycle. The average dose length product of the gated scan of AAA was (897 ± 221) mGy × cm, which did not exceed the published radiation dose in similar papers [12, 13].

## 5. Conclusions

In conclusion, it can be stated that the current method to determine the distensibility of the AAA in subjects gives reproducible results. Functional information from ECG-gated 64-MDCT is of value for the evaluation of aortic distensibility. Taking into consideration the relationship between these different risk factors could lead to a better clinical approach to the AAA patients.

## Figures and Tables

**Table 1:** Clinical characteristics of study population.

Characteristic	Total (*n* = 54)	Male (*n* = 40)	Female (*n* = 14)	*P* value
Age, years	67.2 ± 6.8	58.9 ± 8.3	69.4 ± 7.9	<0.05
BMI, kg/m^2^	29.7 ± 2.9	30.6 ± 3.0	25.8 ± 2.8	<0.05
Heart rate, bpm	73.9 ± 10.1	76.1 ± 12.3	72.9 ± 9.4	NS
Smoking (current) (%)	52% (28/54)	63% (25/40)	21% (3/14)	<0.05^*∗*^
COPD (current) (%)	30% (16/54)	23% (9/40)	50% (7/14)	<0.05^*∗*^
Blood pressure at rest				
Brachial systolic BP, mmHg	136.5 ± 17.7	139.7 ± 19.8	128.5 ± 15.5	NS
Brachial diastolic BP, mmHg	84.3 ± 8.9	87.6 ± 8.2	80.1 ± 8.5	NS
Biochemistry				
Glucose, mmol/L	4.5 ± 0.4	4.3 ± 0.3	4.7 ± 0.7	NS
Cholesterol, mmol/L	4.06 ± 0.8	5.2 ± 1.3	3.7 ± 0.5	<0.05
Triglycerides, mmol/L	0.95 ± 0.4	0.90 ± 0.2	0.99 ± 0.2	NS
HDL, mmol/L	1.41 ± 0.8	1.69 ± 1.1	1.37 ± 0.3	<0.05
LDL, mmol/L	2.4 ± 1.2	2.3 ± 1.0	2.5 ± 1.1	NS

*Note*. Values are percentages or mean ± standard deviation (range).

BMI, body mass index; COPD, chronic obstructive pulmonary diseases; BP, blood pressure; HDL, high-density lipoprotein; LDL, low-density lipoprotein; NS, not significant; SD, standard deviation.

Independent *t-*test unless stated otherwise. ^*∗*^Two-tailed Fisher exact test.

**Table 2:** Distribution of different parameters in 54 patients with AAA.

Parameter	Level 1.	Level 2.	*P* value
Distensibility (10^−5^ Pa^−1^)	1.05 ± 0.22	0.49 ± 0.18	<0.01^*∗*^
Pulse wave velocity (m·s^−1^)	9.68 ± 1.09	14.96 ± 4.01	<0.01^*∗*^

*Note*. AAA, abdominal aortic aneurysm.

Level 1, immediately below the lowest renal artery; Level 2, at the level of maximal aneurysm diameter. ^*∗*^Wilcoxon signed rank test.

**Table 3:** Reliability of distensibility measurements at distinct locations in 54 patients with AAA.

	Level 1.	Level 2.
Intraobserver reliability		
Intraclass correlation coefficient	0.92	0.85
Bland-Altman (10^−5^ Pa^−1^)	0.017 ± 0.15	0.010 ± 0.11
Interobserver reliability		
Intraclass correlation coefficient	0.89	0.79
Bland-Altman (10^−5^ Pa^−1^)	0.013 ± 0.14	0.018 ± 0.08

*Note*. AAA, abdominal aortic aneurysm.

Level 1, immediately below the lowest renal artery. Level 2, at the level of maximal aneurysm diameter.

**Table 4:** Anthropometric and biochemical parameters correlated with aortic distensibility and pulse wave velocity in AAA patients.

Univariate analysis	Aortic distensibility (*D*)		Pulse wave velocity (PWV)	
	*r*	*P* value	*r*	*P* value
Age, years	−0.48	0.006	0.48	0.010
Sex	0.41	0.007	−0.41	0.007
Height, cm	0.28	0.008	−0.28	0.007
BMI, kg/m^2^	−0.32	0.005	0.32	0.006
Brachial systolic BP, mmHg	−0.31	0.005	0.31	0.006
Brachial diastolic BP, mmHg	0.28	0.007	0.28	0.007
Glucose, mmol/l	−0.39	0.005	0.39	0.005
Cholesterol, mmol/l	−0.32	0.005	0.32	0.006
Triglycerides, mmol/l	−0.15	0.003	0.15	0.010
HDL, mmol/l	−0.20	0.004	0.20	0.012
LDL, mmol/l	−0.43	0.007	0.43	0.018
AAA diameter	−0.54	0.008	0.55	0.024

Multivariate analysis	*β*	*P* Value	*β*	*P* Value

Body mass index, kg/m^2^	−0.43	0.006	0.52	0.024
Brachial systolic BP, mmHg	−0.50	0.021	0.53	0.015
AAA diameter	−0.53	0.008	0.55	0.017

*Note*. BMI, body mass index; BP, blood pressure; HDL, high-density lipoprotein; LDL, low-density lipoprotein; AAA, abdominal aortic aneurysm.
